# A Review on Flame Retardants in Soils: Occurrence, Environmental Impact, Health Risks, Remediation Strategies, and Future Perspectives

**DOI:** 10.3390/toxics13030228

**Published:** 2025-03-20

**Authors:** Trang Le Thuy, Tuan-Dung Hoang, Van-Hiep Hoang, Minh-Ky Nguyen

**Affiliations:** 1 Faculty of Environmental and Natural Sciences, Duy Tan University, Da Nang 550000, Vietnam; lethuytrang@dtu.edu.vn; 2 School of Engineering and Technology, Duy Tan University, Da Nang 550000, Vietnam; 3 School of Chemistry and Life Science, Hanoi University of Science and Technology, No. 1 Dai Co Viet, Hai Ba Trung, Hanoi 100000, Vietnam; tuandunghoang@gmail.com; 4 Vietnam National University, Hanoi, VNU Town, Hoa Lac, Thach That District, Hanoi 155500, Vietnam; 5 Hanoi—School of Interdisciplinary Sciences and Arts, Vietnam National University, 144 Xuan Thuy Street, Cau Giay District, Hanoi 100000, Vietnam; 6 Faculty of Environment and Natural Resources, Nong Lam University, Hamlet 22, Linh Trung Ward, Ho Chi Minh City 700000, Vietnam

**Keywords:** brominated flame retardants (BFRs), bioaccumulation, ecological impact, health risk, toxicity

## Abstract

As novel pollutants, flame retardants (FRs) are prone to accumulating in soil and might increase human health risks. It is advisable to emphasize the biomagnification of FRs within the terrestrial food chain, particularly concerning mammals occupying higher trophic levels. Exposure to soil particles laden with FRs may result in numerous health complications. These findings offer significant insights into FR pollutant profiles, tracing origins and recognizing health risks associated with soil samples. Reports have revealed that exposure to FRs can pose serious health risks, including neurodevelopmental impairments, endocrine system disruption, and an increased likelihood of cancer. Nanomaterials, with their high surface area and flexible properties, possess the ability to utilize light for catalytic reactions. This unique capability allows them to effectively degrade harmful contaminants, such as FRs, in soil. Additionally, biological degradation, driven by microorganisms, offers a sustainable method for breaking down these pollutants, providing an eco-friendly approach to soil remediation. These approaches, combined with optimum remediation strategies, hold great potential for effectively addressing soil contamination in the future. Further research should prioritize several key areas, including ecological behavior, contaminant monitoring, biological metabolomics, toxicity evaluation, and ecological impact assessment.

## 1. Introduction

Flame retardants (FRs) have been reported in various environmental settings, including soil, water, air, sediments, organisms, plants, and so on. For instance, tetrabromobisphenol A (TBBPA) has been detected at elevated levels in water, sewage sludge, sediments, soils, air, and other environmental media [1]. FR chemicals are recognized for posing a threat to ecological health. They are present in dust, air, drinking water, and foods, as well as in textiles and indoor surfaces, where they could be absorbed via skin contact. Additionally, they are recognized in natural ecosystems, e.g., oceans, lakes, rivers, sediments, and in fish, mammals, and birds [1,2,3,4]. FRs are chemical substances incorporated into materials to lower their flammability and slow fire spread. To comply with fire safety regulations, these substances are extensively utilized in several products. Brominated flame retardants (BFRs) are commonly applied in plastic-contained products such as electronic devices, toys, textiles, vehicles, building materials, foam beds, and furniture to enhance fire retardancy [5,6]. They are also commonly incorporated into various other consumer products to decrease flammability. However, their environmental persistence and potential health risks have raised alarms. There is increasing apprehension about their efficacy in maintaining fire safety and the possible dangers they present to the environment and human beings.

Due to their low cost and excellent thermal stability, BFRs are extensively applied in chemical, textile, electrical, and construction materials to minimize the risk of fire [7]. The presence of BFR chemicals, especially hexabromocyclododecane (HBCD), polybrominated diphenyl ethers (PBDEs), and TBBPA has raised growing concern among scientists in recent years. Zhang et al. [8] evaluated the concentrations, possible sources, and health threats linked with PBDEs and novel brominated flame retardants (NBFRs) in China’s green belt soil and road dust. The results showed that ΣPBDE levels had a median of 38.19 ng g^−1^ (ranging from 8.07 to 195.33 ng g^−1^) in the green belt soil and a median of 48.01 ng g^−1^ (0.15 to 193.75 ng g^−1^) in the road dust. BFRs are a class of chemicals that have garnered significant attention recently because of their resemblance to “legacy” organohalogenated compounds such as polychlorinated biphenyls (PCBs), particularly regarding their environmental persistence, stability, and accumulation in wildlife and humans [9]. As a result, traditional FRs such as PBDEs and HBCDs are governed by the Stockholm Convention [10].

So far, organophosphate esters (OPEs) and NBFRs have been increasingly used as substitutes to meet flammability regulations. However, numerous potential environmental issues remain that could pose a threat to local residents as these chemicals are persistent pollutants in terrestrial ecosystems. Certain FRs, such as PBDEs, have been prohibited due to their durability, bioaccumulation, and toxicity [11]. This raises critical concerns. Beyond the adverse impacts, remediation strategies are required to protect human health and ecosystems [12,13,14,15]. While various remediation strategies exist, in-depth efforts should focus on developing eco-friendly, cost-effective, and scalable solutions. Continued research on the fate, transformation, and long-term impacts of FRs is crucial for effective pollution management. Hence, this paper focused on FRs in soils and the associated health risks in relation to sustainable development and remediation strategies. The primary objectives of this work were (1) to investigate the occurrence and identify potential sources of soil pollution by FRs, (2) to highlight their environmental impact and health risks, (3) to explore remediation strategies, and (4) to analyze future perspectives and suggest priorities for further research.

## 2. Methods

The review methodology focused on studies examining soil FRs, specifically their occurrence, environmental impact, health risks, remediation strategies, and future perspectives. The review covered an extensive selection of scientific journal articles and academic reports from esteemed publishers, including Scopus, Web of Science (WoS), and Google Scholar, spanning the years 2015 to 2024. Our search incorporated such keywords as “flame retardant”, “FRs”, “brominated flame retardant”, “BFRs”, “soil”, “soil-agriculture system”, “characteristic”, “source”, “occurrence”, ”distribution/abundance”, “environmental impact”, “health risk”, “fate and ecological effect”, “remediation”, and “challenge and perspective” as search terms. At least two authors independently assessed titles and abstracts for inclusion, resolving any discrepancies with the help of a third author. Subsequently, we conducted a full-text review of all the screened articles. Studies were included if they provided experimental data on the environmental effects and health risks of FRs in soil agriculture systems. Data were extracted on the occurrence, impact, health risks, challenges, remediation strategies, and future perspectives of FRs. The findings from the selected studies were synthesized to highlight their characteristics, sources, and occurrence in soil environments, as well as their health impacts and remediation strategies. Initially, 1268 records matching the search parameters were identified. After removing 374 duplicates, the titles and abstracts of 894 papers were reviewed. Among them, 711 studies, including review articles, were excluded for not meeting the selection criteria. Subsequently, 183 full-text papers underwent a detailed evaluation to verify their relevance. Finally, 56 research articles were selected for inclusion in our comprehensive review.

## 3. Characteristics, Sources, and Occurrence in Soil Environments

### 3.1. Characteristics and Sources

FRs, comprising bromine-, chlorine-, and phosphorus-based compounds, are extensively utilized in diverse products or mixtures designed to lower flammability and inhibit or delay fire spread [16,17]. Numerous FRs, especially those with bromine and chlorine, are known for their bioaccumulation and persistence. Conventional BFRs, e.g., PBDEs, exhibit persistent organic pollutant-like traits such as environmental durability, bioaccumulation, biological toxicity, long-range atmospheric transport, etc. [18]. Halogenated FR compounds, including brominated and chlorinated varieties, along with organophosphate flame retardants (OPFRs), constitute nearly 70% of the global market for organic FRs.

In recent years, pentabromodiphenyl ether (penta-BDE), octabromodiphenyl ether (octa-BDE), and decabromodiphenyl ether (deca-BDE) have been widely utilized as commercial BFRs [19,20]. Serving as substitutes for BFRs, OPFRs are extensively utilized in industrial and everyday applications [4]. As PBDEs are included in the list of regulated chemicals, NBFRs have been developed as alternatives and have been widely used since the late 20th century [14]. The significant lipophilicity of NBFRs and PBDEs enables them to accumulate in various aquatic species, bioaccumulate, and biomagnify through the food chain, threatening aquatic ecosystems and human health. Waste disposal is a primary pathway through which PBDEs can be released into the adjacent ecosystem. Known as “legacy BFRs,” PBDEs were once one of the most frequently used FRs globally. PBDEs have been extensively applied as FRs for many years, producing large quantities of waste containing PBDEs [21]. As persistent organic pollutants (POPs) regulated by the Stockholm Convention, tetra- to hepta-BDEs and deca-BDE are characterized by their persistence, toxicity, and bioaccumulation, leading to their regulation in most countries for production and use [22]. These commercial mixtures of PBDEs have also been recognized as POPs under the Stockholm Convention.

**Figure 1:**
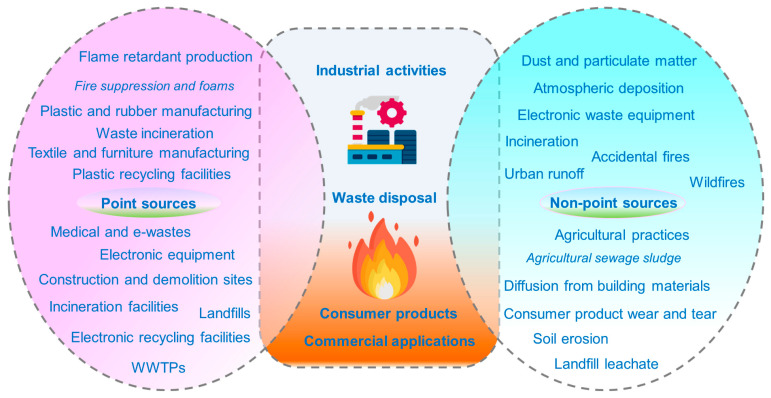
Sources of flame retardants in soils.

The sources of FR pollution are primarily classified into nonpoint and point sources (Figure 1). Point sources of FR contamination mainly include FR production plants, landfill leachate, sewage processing plants, healthcare waste, plastic repurposing units, electronic equipment, waste incineration, and accidental or uncontrolled fires [23,24]. For example, the primary sources of BFRs infiltrating ecological compartments consist of production sites, recycling locations, manufacturing areas, e-waste disposal sites, and wastewater treatment plants (WWTPs) [25]. BFRs are used to manufacture flame-resistant materials for industrial applications, such as electronics, textiles, and plastic equipment. The overuse of plastic items surged significantly throughout the COVID-19 crisis, leading to an increased burden on terrestrial and aquatic environments [26,27]. These items are often disposed of in large quantities, releasing BFRs into various environmental matrices [28,29]. E-waste dismantling is also recognized as a major source of FRs and can potentially harm the surrounding environment [30]. Nonpoint sources include incineration, e-waste devices, soil erosion, dust and airborne particulates, agricultural biosolids, etc. Diffuse sources primarily originate from the extensive utilization of various consumer products. It was also discovered that NBFRs and PBDEs enter ecosystems through various pathways, such as wastewater discharge, surface runoff, atmospheric deposition, and plastic degradation [7,31]. Thus, FRs primarily enter the environment through the following main pathways: (1) during manufacturing and polymer production, (2) throughout the service life of FR-containing products, and (3) during end-of-life management activities, including uncontrolled combustion, mechanical treatment, waste disposal, and incineration.

### 3.2. Occurrence and Distribution of FRs in Soils

Table 1 provides data on the occurrence and spatial distribution of FRs in the soil environment. NBFRs are extensively found in the soils in Australia, China, and Pakistan, with 1,2-bis(2,4,6-tribromophenoxy)ethane (BTBPE), decabromodiphenylethane (DBDPE), 2-ethylhexyl-2,3,4,5-tetrabromobenzoate (TBB), pentabromoethylbenzene (PBEB), and hexabromobenzene (HBB) being the dominant NBFRs in the soil [14]. They were also identified in Antarctic soil, with DBDPE being the primary contaminant in the all samples [18]. The observations demonstrated that the total levels of seven NBFRs varied from 4.89 to 2853 pg g^−1^ d.wt. in the Himalayas [10]. Among the NBFRs, triphenyl phosphate (TPhP) and DBDPE were the primary compounds [10]. Nigerian e-waste recycling sites and dumpsites were marked by a diverse array of OPFRs and plasticizers at elevated µg g^−1^ levels in the soil and outdoor dust [32]. The findings for the samples collected from the soil from dismantling sites and e-waste dumpsites ranged from 1.6 to 62 μg g^−1^ (mean: 1.6 μg g^−1^) and from 0.4 to 42.3 μg g^−1^ (mean: 9.0 μg g^−1^) for ∑17OPFRs in the soil, respectively. OPFRs were linked to both e-waste recycling and automobile dismantling activities. OPFRs exhibit higher water solubility than PBDEs, making them more prone to leaching into groundwater. Once these substances enter groundwater systems, they can remain for years, especially if they are not susceptible to rapid biodegradation. Further, groundwater contamination is challenging to reverse, making it difficult to remediate after contamination has occurred.

Additionally, significant spatial and temporal variations exist due to differences in industrial activities, waste management practices, and environmental conditions. Informal e-waste processing is a significant contributor to soil NBFR contamination. Matsukami et al. [33] noted a swift increase in NBFRs as the levels of PBDEs in the soil surrounding e-waste processing facilities diminished. Throughout the analysis, the PBDE and alternative FR levels in the soils surrounding the e-waste-processing areas varied from 37 to 9.2 × 10^3^ ng g^−1^ d.wt. and from 35 to 2.4 × 10^4^ ng g^−1^ d.wt., respectively. In the soils near the open-burning sites, the concentrations fluctuated from 1.6 ng g^−1^ to 62 ng g^−1^ d.wt. and from < 4 ng g^−1^ to 1900 ng g^−1^ d.wt. [33]. PBDE contamination continues to be elevated in the study area. Their persistence and bioaccumulation potential raise concerns about their environmental risks.

The levels of FRs, such as PBDEs, polybromobenzenes (PBBzs), OPEs, and dechlorane plus, were assessed in the soils of China [30]. The findings revealed that soil pollution was higher in the industrial park compared to the surrounding environment. Despite restrictions under the Stockholm Convention, PBDEs are still detected in soils, particularly near electronic waste (e-waste) sites, landfills, and industrial zones. Their persistence and hydrophobic nature contribute to long-term contamination. In general, there is a growing trend of NBFRs in soils worldwide [14]. These hotspots often exhibit elevated FR levels due to improper disposal and leaching from electronic and plastic waste. Even in the relatively untouched Tibetan Plateau, the total NBFRs in the soil samples gathered in 2019 were notably higher than those determined in 2012 [34]. The level of ∑7NBFRs varied from 34.2 to 879 pg g^−1^ d.wt. in the soil [34]. The occurrence and distribution of NBFRs across various soil types was as follows: “manufacturing land”—5100 ng g^−1^ d.wt., “e-waste disposal area”—4640 ng g^−1^ d.wt., “urban soil”—607 ng g^−1^ d.wt., “farmland”—178 ng g^−1^ d.wt., “forest”—1.52 ng g^−1^ d.wt., and “remote background”—0.85 ng g^−1^ d.wt. [14]. It is crucial to comprehend their emission sources, occurrence, distribution, transformation/bioavailability, toxicity, and mechanisms of action.

**Table 1:** Occurrence and distribution of flame retardants (FRs) in the soil environment.

Location	Media	Flame Retardants	Levels	Remarks	References
China	Forest soil	DBDPE	ND—18,122 pg g^−1^	Contribution of human activities Gas chromatography–mass spectrometry (GC–MS)	[35]
China	Production park and surrounding areas	PBDEs DBDPE	2.88 × 10^4^ ng g^−1^ 8.46 × 10^4^ ng g^−1^	Point source characteristics Originated from human activities Gas chromatography–mass spectrometry (GC–MS)	[36]
Australia	Urban soils	NBFRs	ND—385 ng g^−1^	E-waste recycling and polymer manufacturing are the main sources Gas chromatography–tandem mass spectrometry (GC–MS/MS)	[37]
Nigeria	Dismantling sites E-waste dumpsites	∑17OPFRs	0.2–68 μg g^−1^ (5.5 μg g^−1^) 0.4–42.3 μg g^−1^ (9.0 μg g^−1^)	From e-waste dismantling and dumpsites Liquid chromatography–triple quadrupole mass spectrometry (LC–QQQ)	[32]
Himalayas	Soils, mountain valleys	∑7NBFRs	4.89–2853 pg g^−1^	DBDPE and TPhP were the predominant compounds Gas chromatography–triple quadrupole mass spectrometry	[10]
Nepal	Surface soils	∑HFRs	9.50–3320 ng g^−1^ (median, 144 ng g^−1^)	Long-range atmospheric transport Related to the use of a wide variety of commercial products Gas chromatography–mass spectrometry (GC–MS)	[38]
UK	Surface soils	BDE-209 ΣPBDEs	11 ng g^−1^ 15 ng g^−1^	Urban activity as a source of FRs Gas chromatography–electron ionization–mass spectrometry (GC–EI–MS)	[39]
Brazil	Soils, landfill site	PBDEs NBFRs OPFRs	276 (0.73–851) ng g^−1^ 19 (1.1–83) ng g^−1^ 67 (1.8–186) ng g^−1^	Mismanagement of waste containing FRs Gas chromatography–triple quadrupole mass spectrometry	[40]
Antarctica	Soil	NBFRs	61.2–225 pg g^−1^	DBDPE was the dominant NBFR Gas chromatograph coupled with an electron capture negative ionization mass spectrometer (GC–NCI–MS)	[18]
Italy	Woodland soils	OPFRs and BFRs	0.09–15 ng g^−1^	Environmental contaminants Gas chromatography–triple quadrupole mass spectrometry	[41]

Note: decabromodiphenyl ethane (DBDPE), flame retardants (FRs), halogenated flame retardants (HFRs), brominated flame retardants (BFRs), organophosphorus flame retardants (OPFRs), polybrominated diphenyl ethers (PBDEs), decabromodiphenyl ether (BDE-209), triphenyl phosphate (TPhP), novel brominated flame retardants (NBFRs).

## 4. Environmental Impact and Health Risk

**Figure 2:**
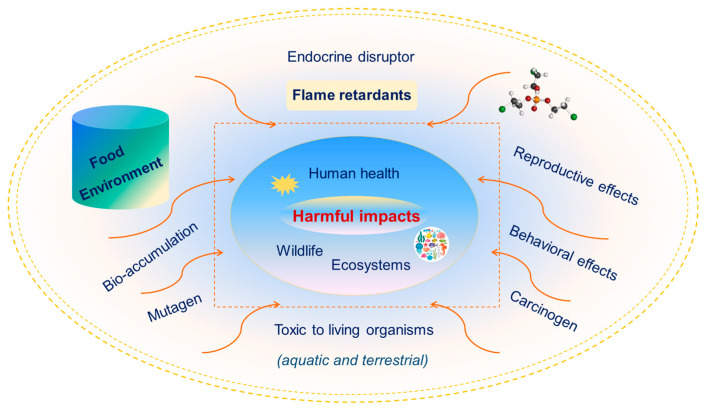
Harmful impact of flame retardants on ecosystems and health risks.

Extensive use of FRs has resulted in serious issues, including their environmental release, the toxic nature of synthetic chemicals, and their contribution to environmental pollution [42]. Their toxicity depends on their chemical composition, persistence, bioavailability, and degradation products. Even FRs with lower toxicity can still pose indirect environmental and health risks due to their accumulation in soil, potential for long-range transport, and transformation into more harmful byproducts. Due to their greater lipophilicity, NBFRs and PBDEs can undergo metabolism and transformation in animals, plants, and humans [31]. Jones et al. [43] identified, organized, and categorized the existing significant evidence of FR substances’ ecologically relevant toxicological impacts on the environment. Numerous studies have demonstrated that OPFRs have various adverse effects, e.g., carcinogenicity, neurotoxicity, and endocrine-disrupting activity [44,45]. This pollution presents ecological risks and can affect human health (Figure 2). 

### 4.1. Ecological and Environmental Impacts

As a result of their large-scale production and use, FRs are considered hazardous, bioaccumulative, and recalcitrant contaminants in diverse environmental settings [28]. Table 2 shows the toxicity effects of FRs on organisms and associated health risks. The occurrence of elevated levels of specific PBDE isomers may lead to harmful effects on wildlife. BFRs recognized as POPs are difficult to break down and are linked to reproductive toxicity and carcinogenicity [8]. Given their extensive use and harmful effects, penta-BDEs and octa-BDEs were designated as priority-controlled contaminants by the “Stockholm Convention for Persistent Organic Pollutants (POPs)” in 2009, and deca-BDE was later included in 2017 [21].

Traditional BFRs, including TBBPA, polybrominated biphenyls (PBBs), and PBDEs, have been shown to be bioaccumulative and to cause a wide array of adverse environmental impacts [9,46]. During the entire lifecycle of these industrial products, there remains a constant risk of PBDEs being emitted into the surrounding environment. Due to their persistence and bioaccumulative nature, these chemicals pose long-term risks to ecosystems. Research has demonstrated that NBFRs and PBDEs, e.g., BDE-99, BTBPE, and HBB, primarily cause apoptosis through oxidative stress, endocrine impairment, and neurodevelopmental toxicity in living forms [11,47].

**Table 2:** Toxicity effects of FRs on organisms and associated health risks.

Flame Retardants	Objects	Toxicity Effects	References
TCEP, TCIPP, TDCIPP	Wheat (*Triticum aestivum* L.)	Oxidative stress Disrupting photosynthesis	[48]
TCIPP	Pakchoi (*Brassica chinensis* L.)	Oxidative stress Growth inhibition Changing chlorophyll and proline content	[49]
EHTBB, TBPH	American kestrels (*Falco sparverius*)	Oxidative stress Thyroid disruption	[50]
TCP	Chicken embryos	Embryonic deformities Impacted growth Altered mRNA expression levels of genes	[51]
TCEP	Rats	Neurotoxicity Memory impairment	[52]
TPhP	Zebrafish (*Danio rerio*)	Development disorders Disrupted neurotransmitter system	[53]
RDP	Zebrafish (*Danio rerio*)	Neurotoxicity	[54]
TCEP, TCP	Earthworm (*Eisenia fetida*)	Neurotoxicity Intestinal damage Oxidative damage DNA damage	[55]
TCP	*Brevibacillus brevis*	Oxidative stress Enhanced cell membrane permeability Disrupted cell membrane	[56]
PBDEs	Human serum	Toxicological concerns	[57]

Note: tricresyl phosphate (TCP), tris(2-chloroethyl) phosphate (TCEP), tris(1-chloro-2-propyl) phosphate (TCIPP), tris(1,3-dichloro-2-propyl) phosphate (TDCIPP), polybrominated diphenyl ethers (PBDEs), bis-(2-ethylhexyl) tetrabromophthalate (TBPH), 2-ethylhexyl-2,3,4,5-tetrabromobenzoate (EHTBB), triphenyl phosphate (TPhP), resorcinol bis(diphenylphosphate) (RDP).

Furthermore, bis-(2-ethylhexyl) tetrabromophthalate (TBPH) and 2-ethylhexyl-2,3,4,5-tetrabromobenzoate (EHTBB) exposure has been demonstrated to cause thyroid function alterations and oxidative stress in birds, fish, rodents, etc. [50]. The results revealed that these FRs could be hazardous to predatory birds. Exposure to EHTBB also leads to oxidative stress and altered thyroid function in American kestrels [50]. Further, BTBPE has been associated with decreased β-galactosidase generation, toxicity, and potential antiestrogenic influences in mammals [58]. BFRs have been identified as important endocrine disruptors, particularly in their capacity to interfere with thyroid hormone-regulated pathways, as shown in in vitro and in vivo investigations. These studies suggest that BFRs interact with thyroid hormone transport proteins, leading to a reduction in thyroid hormone balance in the next generation of exposed animals [9]. Although some research exists, studying NBFRs in soil–agriculture systems is still underexplored and demands more comprehensive investigations.

### 4.2. Health Risk

**Figure 3:**
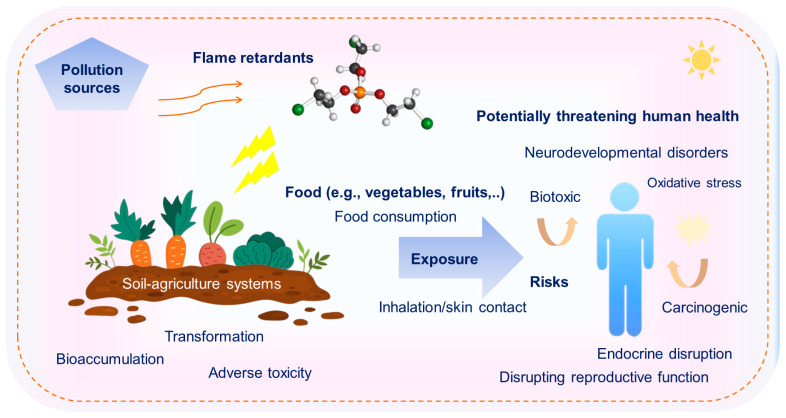
Potentially threatening human health due to FRs in soil–agriculture systems.

The lipophilic nature and high hydrophobicity of PBDEs cause them to accumulate, and they have been recognized in human specimens [57,59]. Figure 3 illustrates the potential threats to human health caused by FRs in soil–agriculture systems. Once in the soil, FRs may alter soil properties, disrupt microbial communities, and be absorbed by crops, leading to potential bioaccumulation in edible plant tissues. The chemical components of OPEs, which have been shown to be biotoxic and carcinogenic, can be released into ecosystems, posing critical threats to human beings [29]. Animal model-based research has illustrated that exposure to PBDEs could disrupt adult male thyroid hormone regulation and reproductive function [46]. Long-term exposure to these chemicals has been linked to adverse health effects. Findings have indicated that exposure to PBDEs could jeopardize human health by leading to neurodevelopmental disorders, endocrine disruption, and cancer [60]. Given their persistence in the environment and bioaccumulative nature, PBDEs continue to pose a significant public health concern, necessitating further research and regulatory efforts to mitigate their impact.

There is also concern that these levels can negatively impact vulnerable human beings, including Indigenous groups, young children, and fish-consuming individuals [7]. Increased concentrations of NBFRs may not only alter the physicochemical profiles of the soil, but could also be absorbed by crops and accumulate in organisms throughout the food web, potentially threatening human health via food consumption and skin contact [14,61,62]. This raises significant concerns for human health, as exposure can occur through the consumption of contaminated food, particularly fruits, vegetables, and animal products. Additionally, direct contact with NBFR-contaminated soil or dust may lead to dermal absorption, further contributing to the body’s toxic burden. Therefore, it is essential to examine the bioaccessibility of micropollutants, their levels, and food materials simultaneously during health risk investigations.

## 5. Strategies for Remediation of Flame Retardants

As stated, the application of FRs is especially worrisome due to their high potential for accumulation, long-term environmental persistence, and tendency to bioaccumulate in ecosystems. Several FRs are of concern due to their possible toxicity to humans and their endocrine-disrupting effects [12]. Strategies for the remediation of FRs in soils to are needed to protect the environment and human health. Various techniques, including photocatalysis-driven oxidation/reduction, adsorption, thermal treatment, and biological processes, have effectively eliminated BFRs from the environment [28]. The documented techniques for remediating PBDE-contaminated soil include biodegradation, phytoremediation, electrokinetic treatment, and electromagnetic methods [28,63,64,65]. Zhou et al. [66] present a detailed review of the methods employed for the elimination of OPEs from soil. For instance, the adsorption method is a commonly employed approach for eliminating OPE-contaminated soil. Several OPEs in soil and on adsorbents have been the focus of research examining their adsorption–desorption processes [66,67]. The sorption isotherm findings indicated that the hydrophobicity of OPEs primarily influenced their affinity for a specific carbon nanotube (CNT), while the π–π electron donor–acceptor interaction also participated significantly in the sorption of aromatic OPEs.

Nanomaterials present a rapidly growing field for eliminating FRs and offer a long-lasting, reliable, and novel solution to removing various types of POPs from the environmental matrices [28]. For example, engineered nanomaterials (such as metal oxides, carbon-based, or polymeric materials) have demonstrated promising performance as absorbents and photocatalysts for removing toxic BFRs. Further, nano zerovalent iron (nZVI) and biochar (BC) are eco-friendly treatment materials used to address pollutants in the environment. In the study of Lu et al. [68], BC-supported nano zerovalent iron (BC/nZVI) particles were prepared from bagasse at 600 °C and subsequently applied to remediate DBDPE in the soil. The outcome showed that BC served as an effective carrier for nano zerovalent iron (nZVI) by reducing accumulation. The maximum remediation performance of BC/nZVI reached 86.9% after 24 h at a mass ratio of BC:nZVI of 2:1, significantly outperforming BC or nZVI alone. DBDPE was initially removed from the soil through adsorption onto BC via hydrogen bonding and π–π interactions. Then, it was oxidized by hydroxyl radicals (•OH) triggered from BC, resulting in the formation of DBDPE-C=O (DP-1) [68]. After that, the debromination of DP-1 occurred gradually as the hydrogen radicals (•H) from the surface hydrolysis of nZVI attacked the bromine atoms on the benzene structure. This solution holds potential as a promising method for remediating soil heavily contaminated with DBDPE. Furthermore, nanomaterials provide a sustainable, efficient, and eco-friendly strategy for removing FRs. The merits of nZVI and nanosized TiO_2_ have been effectively harnessed for in situ treatment of various microcontaminants, including BDE209 [69]. Further, Xie et al. [65] employed nickel/iron bimetallic nanoparticles (dose of 0.03 g g^−1^) for the remediation of 72% of PBDEs from soil. A decline in deterioration efficiency was observed as Ni loading decreased and the initial BDE209 level increased. The developed catalyst remained practical for up to six cycles, demonstrating the sustainability of nanomaterials for repeated practices. 

**Figure 4:**
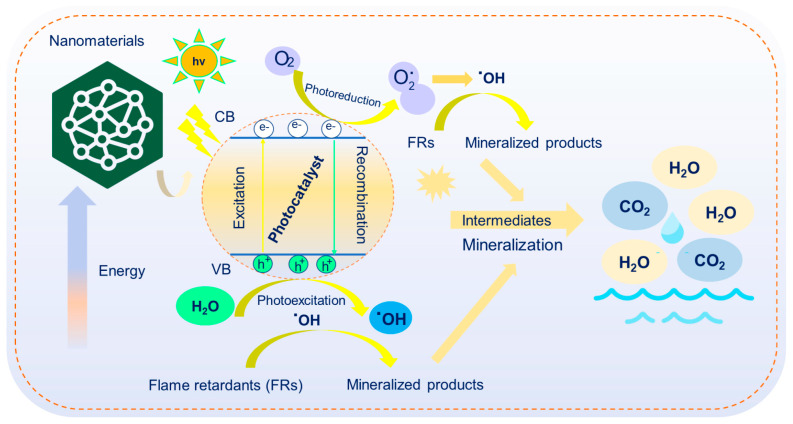
Mechanism of removal of FRs using nanomaterial-based photocatalysts.

In general, both conventional and emerging FRs highlight the essential role of nanomaterials as active photocatalysts in the removal of FRs from soil (Figure 4). Nanomaterials with distinct properties are among the most effective approaches for eliminating FRs through photocatalysis [28]. These methods are highly regarded for their cost effectiveness, speed, and efficiency. Engineered nanoparticles degrade FRs upon exposure to light, transforming them into safer metabolites or fully mineralizing them.

Another approach for eliminating harmful BFRs, particularly HBCD, is through biological degradation. *P. aeruginosa* strain HS9, *Bacillus* sp., *Achromobacter* sp. strain HBCD-2, *Pseudomonas* sp. strain HB01, and *Achromobacter* sp. strain HBCD-1 (these bacteria are capable of breaking down HBCDs) are the microbial sources used for the removal of HBCDs [28,70,71]. The HS9 strain was able to eliminate 69% of 1.7 mg L^−1^ HBCDs within 14 days [70]. Based on the characterization of metabolites, this bacterium was capable of oxidizing HBCDs through two main routes. In the first route, HBCDs were sequentially debrominated to tetrabromocyclododecene (TBCD), dibromocyclododecadiene (DBCDD), and then further debrominated to form cis-, trans-, trans-1, 5, 9-cyclododecatriene. Afterward, cis-, trans-, trans-1, 5, 9-cyclododecatriene was oxidized to 1,2-epoxy-5,9-cyclododecadiene. The second revealed route involves a simultaneous debromination and hydroxylation process, as evidenced by the detection of newly identified 2,5,6,9,10-pentabromocyclododecanols. The results offer valuable insights into the bioremediation of HBCD-contaminated soils.

To investigate the remediation dynamics of the five BFRs (i.e., DBDPE, BDE209, BTBPE, pentabromotoluene (PBT), and HBB) in earthworms, a 7-day test was performed during the 28-day co-exposure period [13]. The contents of these FRs were eliminated over time in earthworms, with the most significant reduction observed after the completion of the exposure period. This offered a theoretical foundation for using earthworms as a biological method for the treatment of FRs. Microorganisms are widespread, highly diverse, and capable of bioremediation by utilizing pollutants as nutrient sources [72]. Yao et al. [23] provided a comprehensive overview of the current advancements in the degradation and removal methods of PBDEs from the environment, including soil. Microbial degradation is widely recognized and offers a sustainable alternative to traditional physical and chemical treatments as it utilizes the metabolic processes of bacteria, fungi, and other microorganisms to degrade PBDEs. Recently, prospective bacterial strains for the bioremediation of FRs were also identified from forest soils in China [35]. The findings demonstrate the potential for applying this approach to soil restoration in areas impacted by long-term exposure to organic pollutants, such as FRs. Microbe-assisted remediation has become a preferred method for the removal of POPs such as BFRs owing to its sustainable and eco-friendly characteristics. This approach takes advantage of the metabolic potential of microorganisms to degrade or transform these harmful pollutants into less harmful or nonhazardous forms, making it a promising alternative to conventional chemical or physical remediation techniques.

## 6. Challenges and Future Perspectives

**Figure 5:**
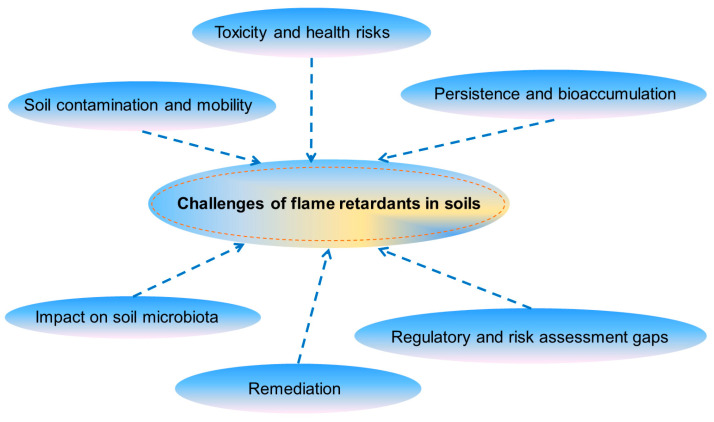
Challenges of flame retardants in soil.

The majority of studies have been conducted in regions with reported contamination, leaving the extent of contamination in unreported areas largely unknown. FRs in soils pose challenges due to their persistence, bioaccumulation, and potential toxicity, impacting soil health, microbial communities, and food safety (Figure 5). Their mobility, analytical complexity, regulatory gaps, and remediation difficulties further complicate effective management and mitigation strategies. The findings suggest that greater focus should be given to the overall risk from multiple contaminants and treatment solutions of FRs in future studies. Knowledge gaps and future research directions should focus on the observation, transformation, toxicokinetics, and ecological risk assessment of NBFRs in aquatic ecosystems. Notably, toxicological data are lacking, particularly regarding in vivo toxicological mechanisms.

1 To better understand their impact on public health, next-phase research is needed to determine the potential health effects linked with chronic and long-term exposure to OPFRs. Future studies should concentrate on investigating the transport pathways of NBFRs between soil and other environmental compartments, as well as on evaluating the cumulative effects of NBFRs on organisms at higher trophic levels [14]. A prospective examination needs to assess the prolonged survival and impacts on organisms exposed to these toxic chemicals.
2 The analysis and monitoring of FRs in soils face numerous challenges, from detection sensitivity and matrix effects to regulatory gaps and long-term environmental variability. Despite progress in research and analytical methods, significant gaps remain in understanding the fate and bioavailability of these pollutants, as well as in developing cost-effective and sustainable remediation technologies. Addressing these challenges will require ongoing interdisciplinary research, improved standardization of analytical methods, and greater collaboration between researchers, regulators, and industry to ensure more effective management of FR contamination in soils.
3 Acute toxicity tests in living forms could not fully elucidate the metabolism and transformation of NBFRs within tissues. Therefore, researchers should focus on the external and internal exposure threats to plants, animals, and humans. To gain a more complete understanding of these chemicals’ behavior and their potential risks, researchers must broaden their focus. Important external exposure can occur through environmental contamination, such as in air, water, and soil, while internal exposure arises from the uptake of these chemicals into the body through ingestion, inhalation, or dermal contact. For example, research should focus on how these chemicals may accumulate in the food chain and impact ecosystems. Understanding these pathways and their subsequent effects on different biological systems is essential for evaluating the full extent of the threat posed by NBFRs.
4 Several techniques, including adsorption, thermal and hydrothermal methods, photocatalytic degradation, reductive debromination, biological degradation, and advanced oxidation processes, have been applied for the elimination of PBDEs [23]. Photocatalysis is among the most commonly reported approaches for PBDE remediation. Nanomaterials, with their unique properties, have proven to be among the most effective approaches for removing BFRs through photocatalysis. Additionally, combining TiO_2_ with other materials such as graphene oxide (GO) or carbon nanotubes (CNTs) can enhance charge carrier mobility and reduce recombination of electron–hole pairs, which significantly improves photocatalytic efficiency. These solutions are widely recognized as cost-effective, rapid, and highly efficient [28]. To enhance the use of these combined processes, further research is needed to simplify their operation and improve the design of integrated approaches. Moreover, biodegradation, particularly microbial degradation, holds promising potential for the remediation of soil contaminated with OPEs [29]. Biological degradation is considered one of the most significant solutions for the removal of PBDEs due to its environmentally friendly nature and low cost [23]. Therefore, to further improve the remediation efficiency, future research could explore combining photocatalysis with biological methods to create a powerful, integrated solution for remediating difficult-to-degrade pollutants such as PBDEs. This hybrid approach would improve pollutant degradation rates, reduce the toxicity of byproducts, and enhance the overall sustainability of the remediation process.
5 The rapid growth of the global population, urbanization, and the impact of the COVID-19 outbreak have made plastic pollution and medical equipment-related waste discharge a substantial global concern [26,73,74]. Some medical devices, protective equipment (e.g., masks and gowns), and electronic medical tools contain BFRs for fire resistance, and their improper disposal during the COVID-19 pandemic likely contributed to BFR pollution in healthcare waste streams. Additionally, incinerating medical plastic waste with BFRs can release toxic brominated compounds into the air, posing environmental and health risks. Recycling is required, and circular economy approaches can help reduce reliance on BFR-containing plastics and mitigate environmental contamination.
6 Comprehending the mechanism of pollutant transfer across phase boundaries is more crucial than simply measuring transfer within a single phase. The root–soil boundary, serving as a transition zone between biotic and abiotic components, represents the primary entry pathway for pollutants into food chains [14]. Further investigation should focus on strengthening the understanding of the mechanisms behind the multiphase transport of NBFRs, particularly the uptake and transfer of FRs from the soil environment by crop roots.
7 Lastly, and equally important, in designing alternative chemicals, one strategy is to prioritize reducing the emission of organic contaminants into the soil and their volatilization into the air. This approach aims to mitigate the environmental and health risks associated with the persistence and spread of harmful substances. An example of a chemical design strategy that reduces the emission of organic contaminants into the soil and their volatilization into the air is the development of bio-based FRs as alternatives to traditional halogenated FRs [75,76]. For future development, efforts should on developing more environmentally friendly and sustainable FR materials.

## 7. Conclusions

Legacy brominated flame retardants (BFRs), illustrated by polybrominated diphenyl ethers (PBDEs), have recently faced tighter regulations due to their environmental durability, bioaccumulation, and adverse toxicity. These pollutants pose significant ecological risks and may adversely impact human health. 

Nanomaterials, particularly in their photocatalytic form, have emerged as promising agents for removing harmful contaminants, such as FRs, from soil. Their high surface area, excellent properties, and ability to utilize light for catalytic reactions make them effective in breaking down persistent pollutants. These nanomaterials can facilitate the degradation of FRs under environmental conditions, offering a sustainable and efficient method for soil remediation. In addition to photocatalytic methods, microbial degradation is widely recognized as a sustainable alternative for addressing soil contamination. This natural process involves microorganisms breaking down complex organic pollutants, particularly BFRs. Recent findings have highlighted the potential of specific microbial strains to degrade these contaminants, effectively offering an environmentally friendly solution. Microbial degradation holds significant promise for remediating soils heavily contaminated with FRs, providing a practical and eco-conscious approach to mitigating long-term environmental damage caused by these persistent pollutants.

Additionally, to overcome emerging challenges, a thorough investigation of the long-term impacts of these processes and the development of practical management approaches are crucial. Environmental pollution from organophosphate (OP) diesters warrants attention, and we anticipate that future research will generate more data to address the existing knowledge gaps in this area. Future research is essential to address the existing gaps and enable a holistic risk assessment of these substances in relation to human and ecosystem well-being.

Current research on flame retardants, such as PBDEs, does not adequately address several key limitations, including the challenges associated with quantitative analysis in soils. The complexity of soil matrices, interference from other contaminants, and low concentrations of flame retardants make detection and accurate quantification difficult. Moreover, the lack of standardized sampling and analytical protocols complicates comparisons across studies and limits the reliability of findings. These issues hinder our ability to fully understand the extent of contamination and the effectiveness of remediation methods.

## Data Availability

The original contributions presented in this study are included in the article. Further inquiries can be directed to the corresponding author(s).

## Abbreviations

BC	biochar
BDE-209	decabromodiphenyl ether
BFRs	brominated flame retardants
BTBPE	1,2-bis(2,4,6-tribromophenoxy)ethane
CNTs	carbon nanotubes
DBCDD	dibromocyclododecadiene
DBDPE	decabromodiphenyl ethane
deca-BDE	decabromodiphenyl ether
EHTBB	2-ethylhexyl-2,3,4,5-tetrabromobenzoate
FRs	flame retardants
GC-MS	gas chromatography–mass spectrometry
GO	graphene oxide
HBB	hexabromobenzene
HBCD	hexabromocyclododecane
HFRs	halogenated flame retardants
NBFRs	novel brominated flame retardants
nZVI	nano zerovalent iron
octa-BDE	octabromodiphenyl ether
OPEs	organophosphate esters
OPFRs	organophosphate flame retardants
PBBs	polybrominated biphenyls
PBBzs	polybromobenzenes
PBDEs	polybrominated diphenyl ethers
PBEB	pentabromoethylbenzene
PCBs	polychlorinated biphenyls
penta-BDE	pentabromodiphenyl ether
POPs	persistent organic pollutants
RDP	resorcinol bis(diphenylphosphate)
TBB	2-ethylhexyl-2,3,4,5-tetrabromobenzoate
TBBPA	tetrabromobisphenol A
TBCD	tetrabromocyclododecene
TBPH	bis-(2-ethylhexyl) tetrabromophthalate
TCEP	tris(2-chloroethyl) phosphate
TCIPP	tris(1-chloro-2-propyl) phosphate
TCP	tricresyl phosphate
TDCIPP	tris(1,3-dichloro-2-propyl) phosphate
TPhP	triphenyl phosphate
WWTPs	wastewater treatment plants
